# From Pathogenesis, Clinical Manifestation, and Diagnosis to Treatment: An Overview on Autoimmune Pancreatitis

**DOI:** 10.1155/2017/3246459

**Published:** 2017-01-19

**Authors:** Ou Cai, Shiyun Tan

**Affiliations:** Department of Gastroenterology, Renmin Hospital of Wuhan University, Wuhan, Hubei, China

## Abstract

Autoimmune pancreatitis (AIP) is a special type of chronic pancreatitis which is autoimmune mediated. The international consensus diagnostic criteria (ICDC) 2011 proposed two types of AIP: type I is associated with histological pattern of lymphoplasmacytic sclerosing pancreatitis (LPSP), characterized by serum IgG4 elevation, whereas type 2 is named idiopathic duct-centric pancreatitis (IDCP), with granulocytic epithelial lesion (GEL) and immunoglobulin G4 (IgG4) negative. The pathogenic mechanism is unclear now; based on genetic factors, disease specific or related antigens, innate and adaptive immunity may be involved. The most common clinical manifestations of AIP are obstructive jaundice and upper abdominal pain. The diagnosis can be made by a combination of parenchymal and ductal imaging, serum IgG4 concentrations, pancreatic histology, extrapancreatic disease, and glucocorticoid responsiveness according to ICDC 2011. Because of the clinical and imaging similarities with pancreatic cancer, general work-up should be done carefully to exclude pancreatic malignant tumor before empirical trial of glucocorticoid treatment. Glucocorticoid is the most common drug for AIP to induce remission, while there still exists controversy on steroid maintenance and treatment for relapse. Further studies should be done to identify more specific serum biomarkers for AIP, the pathogenic mechanisms, and the treatment for relapse.

## 1. Introduction

Autoimmune pancreatitis (AIP) is a special form of chronic pancreatitis that is autoimmune mediated [1]. Autoimmunity is defined as acquired immune reactivity against self-antigens. Autoimmune diseases (AIDs) occur when autoimmune responses lead to tissue damage. AIDs are often classified into two patterns; some are organ specific, for example, diabetes mellitus, in which the pancreas is the target organ, whereas others are systemic, for example, systemic lupus erythematosus (SLE), in which many tissues and organs of the body are damaged. Some common AIDs include diabetes mellitus type 1, Grave's disease, multiple sclerosis, psoriasis, rheumatoid arthritis, and SLE. AIP belongs to and shares some characteristics with AID in pathophysiology, clinical manifestations, and treatment and of course has its uniqueness. The prevalence rate of AIP in Japan was 4.6 per 100,000 individuals in 2011 and the annual incidence rate was 1.4 per 100,000 individuals [2]. In 1961, Sarles et al. [3] first reported a case about nonalcoholic chronic pancreatitis accompanied by hypergammaglobulinemia and predicted its association with an autoimmune process. In 1995, Yoshida et al. [4] first proposed the clinical entity of autoimmune pancreatitis. From then on, more and more scholars have paid attention to this rare type of chronic pancreatitis and substantial progress has been made in the recognition of AIP. The international consensus diagnostic criteria (ICDC) 2011 [5] proposed two forms of AIP: type I is associated with histological pattern of lymphoplasmacytic sclerosing pancreatitis (LPSP), accompanied with the serum immunoglobulin G4 (IgG4) elevation, whereas type 2 is characterized by idiopathic duct-centric pancreatitis (IDCP), with granulocytic epithelial lesion (GEL) and IgG4 negative [5, 6]. The diagnosis of AIP depends on serum IgG4 concentration, pancreatic histology, pancreatic parenchymal and duct imaging, other organ involvement, and steroid reaction and is most often confused with pancreatic cancer, especially the focal AIP exhibiting mass formation [5, 7, 8]. Therefore, some patients with focal AIP have undergone surgical resection due to the suspicion of malignancy, despite recent improvements in radiological imaging modalities [9–13]. Kobayashi et al. [8] reported 11 (72.2%) AIP patients had undergone surgery due to a preoperative diagnosis of mass formation pancreatitis with possible cancer revealed to be focal AIP. Hence, we sought to prepare an updated review about AIP to get a comprehensive knowledge about it.

## 2. Classification

The international consensus diagnostic criteria (ICDC) 2011 [5] had classified AIP into two types. Type 1 called lymphoplasmacytic sclerosing pancreatitis (LPSP), or without granulocyte epithelial lesions (GELs), has some characteristic features in histopathology: dense infiltration of plasma cells and lymphocytes; peculiar storiform fibrosis; obliterative phlebitis [15]; elevated IgG4-positive plasma cells (generally >50 cells per high-power field [HPF] [16]). It generally is believed to be the pancreatic manifestation of an IgG4 related systematic disease and is often accompanied with some extrapancreatic lesions, such as sclerosing cholangitis, sclerosing sialadenitis, and retroperitoneal fibrosis [5, 15, 17]. This type of AIP usually presents with obstructive jaundice in elderly male subjects and responds well to steroid therapy [2, 5, 18].

Type 2 called idiopathic duct-centric pancreatitis (IDCP) has the unique characteristic feature of intraluminal and intraepithelial neutrophils in medium-sized and small ducts as well as in acini in histopathology, which is not seen in LPSP [5]. Also, they share some features in histopathology, such as periductal lymphoplasmacytic infiltration and storiform fibrosis. IDCP often has no or few IgG4-positive cells (<10 cells/HPF) and it seems to be a pancreatic-specific disorder, because it is IgG4 negative and is not associated with other organ involvement (OOI) [5]. Patients in IDCP are often a decade younger and do no show gender preference. IDCP lacks a serological marker and for its diagnosis pancreatic histology is a must [5]. The comparisons between the two types of AIP are in Table 1.

## 3. Pathogenesis

Recent studies have suggested several possible pathogenic factors in the development of AIP, though its pathogenic mechanism remains unclear. Based on genetic factors, disease specific or related antigens, innate and adaptive immunity may be involved [19].

### 3.1. Genetic Factors

Kawa et al. [20] first revealed that the susceptibility of AIP in Japanese patients may be associated with class II antigen haplotype of the major histocompatibility complex (HLA-DRB1^*∗*^0405-DQB1^*∗*^0401). Later, Umemura et al. found that serum IgG4 concentrations in Japanese patients with AIP were significantly positively correlated with the number of susceptible Fc receptor-like 3 (FCRL3) genes alleles [21] expressed on B cells in 2006 and cytotoxic T lymphocyte antigen 4 (CTLA-4) [22] expressed on CD4^+^ and CD8^+^ T cells in 2008. In 2011, Ota et al. evaluated the association of AIP with single nucleotide polymorphisms (SNPs) and provided the evidence of KCNA3 [23] association with AIP. Chang et al. revealed the association of cystic fibrosis transmembrane conductance regulator (CFTR) gene variants [24] with AIP. Although the functions of the CFTR variants and their roles in the pathogenesis of AIP were not elucidated that clear, CFTR variants may play roles as disease modifiers in AIP (seen in Table 2). Undeniably, FCRL3 is found to be associated with various autoimmune diseases, such as rheumatoid arthritis, autoimmune thyroid disease, and SLE in Japanese populations [25, 26].

### 3.2. Immunogenic Factors

AIP is an autoimmune-mediated disease and abnormal immune response may play an important role in its pathophysiology. More than one autoantibody is seen in AIP patients and some other antigens like lactoferrin (LF), carbonic anhydrase (CA) II [27, 28], pancreatic secretory trypsin inhibitor (PSTI) [29], amylase alpha 2A [30], and type IV collagen [31] may also be involved in the pathogenesis of AIP. While combining amylase alpha 2A with IgG4 in diagnosing AIP, the specificity can be 99%, higher than the specificity of 96% while using IgG4 only in a clinical study [32].

As for innate immune response, Watanabe et al. reported that activation of toll-like receptors (TLRs) and nucleotide-binding oligomerization domain- (NOD-) like receptors (NLRs) in monocytes and basophils of patients with IgG4 related disease (IgG4-RD) induced IgG4 production by B cells via B cell activating factor (BAFF) [33, 34]. What is more, Fukui et al. reported that abundant infiltration of TLR-7 positive M2 macrophages was observed in the pancreatic tissues in type 1 AIP patients [35].

As for adaptive immune response, B cells and T cells are unavoidable topics. A recent study showed that increased CD19^+^CD24^high^CD38^high^ regulatory B cells (Bregs) might suppress the disease activity of type 1 AIP, while the decreased CD19^+^CD24^high^CD27^+^ Bregs may be involved in the development of type 1 AIP [36]. Circulatory naïve regulatory T cells (Tregs) are significantly decreased in peripheral blood, while memory T cells are significantly increased in type 1 AIP patients [37]. In addition, prominent infiltration of Tregs with upregulation of IL-10 is observed in the liver of type 1 AIP patients [38]. Li et al. found significant CD8^+^ T lymphocyte infiltration in the pancreas and extrapancreatic lesions in a case of AIP misdiagnosed as pancreatic cancer, indicating that CD8^+^ T lymphocyte might have some effect on the cause of AIP [39].

## 4. Clinical Manifestation

The clinical manifestations of AIP are complex and lack of specificity; therefore, it is extremely difficult to diagnose AIP from symptoms only. Type 1 AIP is typically diagnosed later in life (the mean age at diagnosis is older than 60 years) [2, 18]. Obstructive painless jaundice and upper abdominal pain are the most common complaints. Other rare symptoms include body weight loss, general fatigue, and even no symptoms [40]. A series of studies have been focused on the symptoms and treatments of AIP in different countries and have got different results [41–44] (shown in Table 3). A retrospective study from China showed that the jaundice accounted for 72% and abdominal pain was 44% [41]. Another multicenter study in Spain indicated that abdominal pain accounted for 65.7% and obstructive jaundice was 51.9% in AIP patients [44]. Ueki et al. [45] reveal that type 2 AIP can have the symptoms of acute, constant abdominal pain like in acute pancreatitis, different from the character of chronic pancreatitis.

Besides, AIP can cause extrapancreatic lesions including sclerosing cholangitis, retroperitoneal fibrosis, lachrymal and salivary gland lesions, pulmonary lesions including hilar lymphadenopathy, and tubulointerstitial nephritis, hypophysitis, chronic thyroiditis, and prostatitis [5, 15, 17, 40, 46–50] and biliary tract is the most commonly involved extrapancreatic site [43, 46], which probably explains why there is painless jaundice in AIP patients.

AIP has certain comorbidities. Finkelberg et al. [51] reported that, in AIP patients, approximately 50% have diabetes. More and more attention is focused on the relationship between AIP and inflammatory bowel disease, and the prevalence of IBD in patients with AIP seems to be increased compared to the general population, with 6 to 27% of AIP patients having concomitant IBD [45, 52, 53], especially in type 2 AIP.

## 5. Diagnosis

### 5.1. Diagnosis Criteria for AIP

In 2002, the Japan Pancreas Society (JPS) [54] first proposed the diagnostic criteria for AIP and made the image abnormal findings such as irregular narrowing of the main pancreatic duct (MPD) (>one-third of the entire pancreas) and parenchymal swelling as necessity, accompanied with either of the following two: (1) serology showing hypergammaglobulinemia (>2 g/dL, autoantibodies) and serum IgG elevation (>1800 mg/dL) and (2) characteristic pathological findings including lymphoplasmacytic infiltration with fibrosis. The JPS criterion was revised in 2006 [55], and it first proposed the IgG4 elevation as the serology finding, which is important for the diagnosis of AIP even until now. Because of the limitations in JPS, 2006 [55], Korean Kim criteria [56] occurred, including four parts of imaging, laboratory examinations, histology, and steroid effect. Subsequently, HISORt [14, 57] (based on the four parts in Korean criteria, other organ involvement was added), Asian [58] (histology only can be used to diagnose AIP when it meets the demand), and Manheim criteria [59] have been proposed around the world. In 2011, Shimosegawa et al. [5] first proposed the ICDC for AIP, which is the most accepted major diagnostic criterion. Later, JPS 2011 [60, 61] was proposed in response to the ICDC's inclusion of response to steroid treatment.  Table 4 shows the comparisons of diagnostic criteria in different countries.

There are several diagnostic criteria for AIP in different countries, but ICDC is the first universally accepted criterion of AIP because it considers ethnic and region differences and classifies AIP into two subtypes. The diagnosis of AIP includes five dimensions: serology, histology, imaging, other organ involvement, and steroid effect.

### 5.2. Serology Changes in AIP

Since being proposed by JPS 2006, serum IgG4 elevation is widely used in the diagnosis of AIP. However, IgG4 has its limitations. Studies have shown that 4–10% of both healthy controls and controls with other diseases have high serum IgG4 concentrations [62–64]. In addition, about 20% of patients with AIP have normal serum IgG4 concentrations at presentation [63, 65]. A systematic review with meta-analysis about IgG and IgG4 shows that the pooled sensitivity of serum IgG4 was 0.74 and the pooled specificity was 0.94 [66]. An ideal serological marker should be both sensitive and specific, while IgG4 is neither sufficiently sensitive nor specific. Besides, elevated IgG4 is seen only in type 1 AIP whereas type 2 AIP often has normal IgG4 level. Considering these two factors, searching for new serological marker is essential and valuable. Song et al. [67] proposed combining measurement of serum IgG and IgG4 instead of IgG4 alone to increase the sensitivity in diagnosing AIP. Recently, Hao et al. [68] explored that hybrid kappa (*κ*)/lambda (*λ*) antibody, which composes a substantial portion of IgG4 in normal human serum and is formed by two IgG4 heavy chains plus one *κ* and one *λ* light chain, is a new serological marker for diagnosing AIP. The sensitivity and specificity of hybrid *κ*/*λ* antibody were 80.3% and 91%, respectively. While combining serum IgG4 and the hybrid *κ*/*λ* antibody, the diagnostic sensitivity could be increased from 78.7% to 90.2% compared with serum IgG4 alone without sacrificing specificity significantly.

### 5.3. Imaging Features of AIP in Different Examinations

Ultrasound (US) is widely used for its noninvasiveness, low price, and easy operation. US can present the diffuse enlargement and hypoechoic pancreas, but it cannot show the irregular narrowing or stenosis of the pancreatic duct. Quantitative perfusion analysis in pancreatic contrast enhanced ultrasound (DCE-US) can show the vascular lesions of pancreas and play a significant role in differentiating AIP from pancreatic cancer [69].

Computed tomography (CT) is the most important tool to diagnose AIP and distinguish it from pancreatic cancer. The typical image finding is diffuse morphological pancreatic parenchymal enlargement and the atypical findings include focal enlargement of the pancreas, no enlargement or normal pancreas, and mixed patterns [57, 70–73]. AIP demonstrates a diminished pattern of enhancement in the arterial phase and a relatively increased or prolonged enhancement in the delayed or venous phase [72, 74]. And a capsule-like low density rim is a distinctive finding on CT in AIP [72]. However, if there is low density mass on contrast enhanced CT, pancreatic cancer should be considered.

Magnetic resonance imaging (MRI) has advantages over CT on the capsule-like imaging of the pancreatic duct and surrounding lesions, which is the result of the fibrosis of the pancreas. The typical MRI findings include hypointense signal on T1 weighted images and relatively T2 hyperintense signal [75]. Diffusion weighted imaging (DWI) has been increasingly utilized as a MRI sequence for evaluating pancreas [76–78]. Kim et al. [79] found that while perfusion fraction (*f*) is 0.933, it is most useful for differentiating AIP and normal pancreas and its sensitivity is 85.7% and specificity is 100%. And perfusion fraction (*f*) and perfusion-related diffusion coefficient (*D*_fast_) are more useful than pure molecule diffusion coefficient (*D*_slow_) in differentiating pancreatic diseases from normal pancreas.

Magnetic Resonance Cholangiopancreatography (MRCP) is widely used for its advantage of high quality image and noninvasiveness, but for its less sensitivity in the focal form of AIP and pancreatic cancer, it cannot replace ERCP completely. MRCP could show the diffused narrow or segmental stenosis of main pancreatic ducts, the pancreatic segment of common bile duct stricture, proximal bile duct dilation, and gallbladder enlargement [80].

Endoscopic retrograde cholangiopancreatography (ERCP) is an invasive method but it is feasible in treatment and diagnosis of AIP and the incidence of ERCP-related adverse events is low in patients with type 1 AIP [81]. Ductal imaging, ERCP, may show a long, narrow ductal stricture, or multiple, noncontinuous strictures without marked upstream dilation, and side branches arising from the stricture [82, 83]. The multicenter study carried out by Sugumar et al. [84] has highlighted that the ability of ERCP to diagnose AIP based on ERCP feature alone is limited, but taken together with clinical symptoms, serology, and/or histology it can be useful.

Endoscopic Ultrasound (EUS) can be utilized to evaluate the pancreatic parenchyma, bile duct, and pancreatic duct, as well as in evaluating the bile duct stricture. The EUS guided fine needle aspiration (EUS-FNA) is not included in ICDC as a method for histopathologic diagnosis of AIP because of the difficulty in obtaining adequate specimens for histological analysis. Although EUS-FNA has its limitations for 20.5% unsuccessful adequate tissue sampling, 23 of the 53 undetermined patients could be diagnosed as definitive type 1 AIP without the aid of pancreatic imaging, serology, other organ involvement, and response to steroids [84], which is unique. The nationwide epidemiology survey of AIP in Japan in 2011 found that the use of EUS-FNA increased to 63.8% from 48.4% and the utility of EUS-FNA for establishing of AIP will be further validated in the future [2, 85–87].

Positron Emission Computed Tomography (PET) can get the total image of every part of the body and it is especially sensitive in finding tumors. PET is more sensitive than conventional imaging to detect organ involvement and uptake of fluorodeoxyglucose in organs other than the pancreas often suggests AIP when the clinical characteristic, histology, and serum detection incline the diagnosis of IgG4 related disease [88, 89].

Every imaging method has its cons and pros (shown in Table 5). What should be emphasized is that methods are not isolated; we can combine two or more methods when needed. Uchida et al. [90] stated that in their institution they initially use CT scans to evaluate the enlarged pancreas followed by evaluation of the main pancreatic duct by ERP. For pancreatic head lesions with obstructive jaundice or biliary enzyme abnormality due to biliary stricture, they first perform diagnostic and therapeutic ERCP. For pancreatic head lesions without obstructive jaundice, they perform EUS-FNA followed by diagnostic and therapeutic ERCP. For pancreatic body or tail lesions, they first perform EUS-FNA.

## 6. Differential Diagnosis and the Strategy for Distinguishing AIP from Pancreatic Cancer

As a new and relatively rare pancreatic lesion, AIP is easy to be neglected and misdiagnosed as pancreatic cancer for its clinical and imaging features. As is proposed in ICDC, IgG4 elevation is a high-specific serum marker for AIP [49, 50]. However, Ngwa et al. [91] reported that 10.1% of 548 patients with pancreatic cancer have elevated serum IgG4, which may be confusing when serum IgG4 is used to differentiate pancreatic cancer and AIP. Serum CA19-9 was stated to be useful for distinguishing AIP from pancreatic cancer [92], while CA19-9 can also be elevated in other pancreatic diseases or in other pathological states [93]. Thus, so far, a simple serological marker for the differential diagnosis of AIP from pancreatic cancer is still lacking. What is worse, the differentiation by imaging also presents some problems, especially pancreatic cancer and the focal AIP exhibiting mass formation [7, 8]. Thus, a thorough work-up is essential before either surgery or steroid treatment is planned.

**Figure 1:**
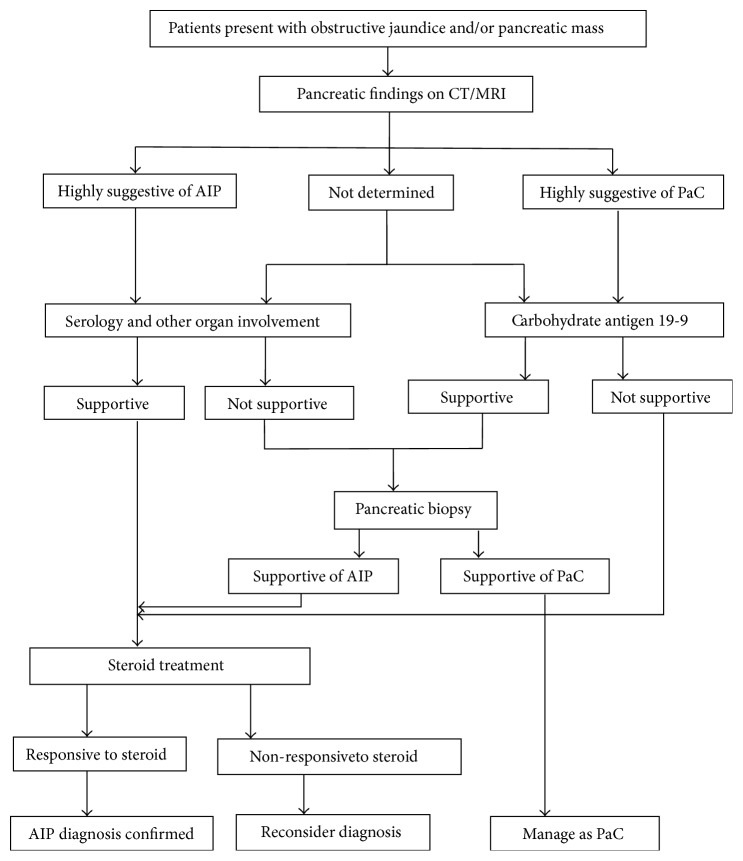
Strategy for distinguishing AIP from pancreatic cancer [14]. CT: computed tomography; MRI: magnetic resonance imaging; PaC: pancreatic cancer; OOI/O: other organ involvement; S: serology; CA19-9: carbohydrate antigen 19-9.

Here we present the American diagnostic strategy to differentiate AIP from pancreatic cancer. In patients with obstructive jaundice and/or pancreatic mass CT findings typical for AIP, the presence of any collateral evidence for AIP (elevated IgG4 or autoantibodies or other organ involvement) is sufficient to make the diagnosis. On the other hand, those with any of the features highly suggestive of pancreatic cancer should generally be managed as cancer unless there is clear evidence of other organ involvement suggestive of AIP. Patients without typical findings of AIP, including those with indeterminate CT findings, should undergo work-up for cancer. If negative, additional collateral evidence for AIP (serum IgG4) should be sought. Diagnosis of AIP is confirmed by pancreatic core biopsy, steroid trial and surgical resection [14] (Figure 1).

## 7. Treatment for AIP

Glucocorticoids are the routine drug for AIP and rapid response to steroid treatment is one of the primary characteristics of AIP. A poor response to steroid therapy might suggest misdiagnosis, especially in the case of pancreatic cancer. Hart et al. [18] conducted a multicenter, international analysis (1064 patients), showing that 99% of type 1 AIP and 92% of type 2 AIP got clinical remission after steroid treatment. Before induction of remission by an initial steroid therapy, management of blood glucose and biliary drainage is recommended in patients with diabetes mellitus and obstructive jaundice. Generally, patients are given initial oral prednisolone dose of 0.6 mg/Kg/day for induction of remission, which is administered for 2 to 4 weeks. The dose is then tapered by 5 mg every 1 to 2 weeks to a maintenance dose (2.5–5 mg/day) that should be continued for three years as maintenance therapy in Japan [94] and the regimen is utilized in most Asian countries. In a multicenter study in Japan, Kamisawa et al. [95] reported that relapse occurred significantly less during maintenance steroid therapy than after the discontinuation of therapy (23% versus 34%, *P* < 0.05), while in European and American countries, maintenance therapy is not commonly used. Ghazale et al. [96] conducted a study and the initial steroid regimen was as follows: prednisolone 40 mg/day orally for 4 weeks, then tapering by 5 mg/week until 11 weeks, and then having a maintenance dose from 12 weeks (Table 6). As a result, 16 (53%) of 30 patients associated with sclerosing cholangitis relapsed during a median follow-up of 29.5 months. Moreover, long time maintenance steroid therapy may cause steroid-related side effects and not all patients can tolerate them. Therefore, whether a maintenance therapy is needed or not needs international discussion.

In a multicenter study in Japan, the cumulative rate of relapse after starting steroid therapy was 56% at 1 year, 76% at two years, and 92% after 3 years [95]. Hart et al. [18] found the relapse was related to IgG4 related sclerosing cholangitis, no business with serum IgG4 level or pancreatic parenchyma involvement (diffuse or focal pancreatic parenchyma enlargement), while Shimizu et al. [97] confirmed that the rate of decrease in serum IgG4 level was significantly higher in nonrelapse group than in the relapse group after steroid treatment and it might be a predictor of a relapse of AIP. The treatment for relapse is restarting steroids. Whether or not to use alternative immunosuppressant, such as azathioprine, methotrexate, and mycophenolate mofetil [5, 18], depends on the patient's reaction to re-steroid therapy and his tolerance to steroid. Unfortunately, in some cases, the patients cannot tolerate both steroid and immunosuppressant and require drug discontinuation. Rituximab, a monoclonal CD20 antibody, has been shown to be useful in treating AIP patients [98, 99]. The effectiveness of rituximab shed light on the role of B cells in the pathogenesis of AIP because of the B cell depletion. As rituximab is the only drug for induction of remission other than glucocorticoids, it would be extremely useful in patients who are unable to tolerate high-dose corticosteroids, require high doses of prednisolone to maintain remission, or have failed to respond to immunomodulator therapy. Currently approved for treating B cell lymphoma and rheumatoid arthritis, rituximab's approval for treating AIP will come true.

The prognosis of AIP is good in general and the long-term complication is rare. Pancreatic duct stones and canceration are the main sequelae [18]. Kanno et al. [2] conducted the nationwide epidemiological survey in Japan in 2011 and found that during the course of observation (1623.3 days), malignant tumors were detected in 109 of 923 patients (11.2%). Shiokawa et al. [100] reported that AIP patients had a high risk of having various cancers, while Hart et al. [101] reported that cancer risk of AIP patients was similar to that of control subjects. Whether AIP is the risk factor for developing cancer needs further investigation.

## 8. Conclusion

In conclusion, AIP is a special type of chronic pancreatitis, whose pathogenic mechanism, maybe a combination of genetic factors and immunity abnormality, needs more work to be clarified. The diagnosis of AIP depends on serology, imaging, histology, other organ involvement, and reaction to steroids, while high sensitive serum biomarkers for AIP subtypes lack. AIP reacts well to steroids, but controversy exists on the steroid maintenance and treatment for relapse. For AIP shares similarity with pancreatic cancer in clinical and imaging characteristics, general work-up is necessary to differentiate them. Future research may be focused on the pathogenesis, the novel serum biomarker, and the relapse treatment for AIP.

## Figures and Tables

**Table 1:** Comparisons of the two types of AIP.

Characteristics	Type 1	Type 2
Other nomenclatures [5]	LPSP	IDCP
	AIP without GEL	AIP with GEL
	IgG4 related	IgG4 unrelated

Ethnic [5]	Asia > United States, Europe	Europe > United States > Asian

Age [2, 5, 18]	60 years or older	A decade younger

Sex [5]	Usually male	Equal

Symptom [5]	Obstructive jaundice often	Obstructive jaundice often
	Abdominal pain rare	Abdominal pain common
	Pancreas swelling common	Pancreas swelling common

Serology [2, 5]	High serum IgG4, auto-Ab+	Normal serum IgG4, auto-Ab−

Histopathology [5]	Lymphocyte and plasmacyte infiltration and fibrosis	Granulocyte epithelial lesion often with destruction and obliteration of the pancreatic duct
	Infiltration of IgG4 plasma cells	

Extrapancreatic lesion [5, 15, 17]	Sclerosing cholangitis	Unrelated with OOI
	Sclerosing sialadenitis	
	Retroperitoneal fibrosis, etc.	

Ulcerative colitis [2, 5]	Rare	Often

Histology needed for diagnosis [5]	No	Yes

Respond to steroid [2, 5]	Responsive	Responsive

Relapse rate [5]	High	Low

**Table 2:** Genetic factors in the pathogenesis of AIP.

Gene related	Cells involved	Sites related	Possible function in AIP	Referencing
HLA-DRB1^*∗*^0405-DQB1^*∗*^0401	T cells	HLA-DRB1^*∗*^0405-DQB1^*∗*^0401 haplotype	Inducing an autoimmune response; genetic marker for non-HLA gene associated disease susceptibility	Kawa et al. [20]

FCRL3	B cells	FCRL3-110 alleles	Susceptibility with AIP	Umemura et al. [21]

CTLA4	T cells	+6230G/G +49A/A	Being related with AIP resistance; marker of risk of relapse in AIP	Umemura et al. [22]

KCNA3	T cells	SNP (rs2840381, rs1058184, rs2640480, rs1319782)	T cell proliferation and activation	Ota et al. [23]

CFTR	—	Variants (1556V, 5T, S42F, etc.)	Predictors of a slow and reduced response to steroid treatment in AIP	Chang et al. [24]

**Table 3:** Symptoms of AIP in different studies.

Year	Number of patients	Ethnic	Male : female	Jaundice	Abdominal pain	Weight loss	No symptoms
2008 [41]	25	Chinese	22 : 3	18 (72%)	11 (44%)	10 (40%)	3 (12%)
2011 [42]	731	8 countries	—	Type 1 AIP 75%	Type 1 AIP 41%	—	—
				Type 2 AIP 47%	Type 2 AIP 68%		
2015 [43]	705	Chinese	4.47 : 1	63.4%	62.3%	45.1%	2.9%
2016 [44]	52	Spain	—	27 (51.9%)	34 (65.4%)	—	—

**Table 4:** Comparisons of diagnostic criteria in different countries.

Diagnostic criteria	Japanese criteria(2006) [55]	SIHORts (2006) [57]	Korean criteria (2007) [56]	Asian criteria (2008) [58]
A: imaging	Diffuse or segmental narrowing of the MPD; diffuse or localized enlargement of the pancreas	Typical imaging features: diffusely enlarged gland with delayed (rim) enhancement; diffusely irregular and attenuated MPD Atypical imaging features: focal pancreatic mass, focal pancreatic duct stricture	Diffuse enlargement of pancreas and diffuse or segmental irregular narrowing of MPD	Typical imaging features: diffusely enlarged gland with delayed (rim) enhancement; diffusely irregular and attenuated MPD Atypical imaging features: focal pancreatic mass, focal pancreatic duct stricture

B: serology	High serum *γ* globulin, IgG, IgG4, or the presence of autoantibodies	Elevated serum IgG4 level	Elevated levels of IgG and/or IgG4 or detected autoantibodies	High level of serum IgG or IgG4 or detected autoantibodies

C: histology	Infiltration of lymphocytes and plasma cells	Lymphoplasmacytic infiltrate with storiform fibrosis showing abundant	Fibrosis and lymphoplasmacytic infiltration	Lymphoplasmacytic infiltration with fibrosis, with abundant IgG4-positive cell infiltration
		(>10 cells/HPF) IgG4-positive cells		

D: other organ involvement	Not included	Biliary stricture, parotid/lacrimal gland involvement, mediastinal lymphadenopathy, retroperitoneal fibrosis	Included	Not included

E: steroid effect	Not included	Included	Included	Included

Definite diagnosis	Criterion A + B Criterion A + C	Criterion A + B Criterion A + C Criterion A + D Criterion A + E	Criterion A + B Criterion A + C Criterion A + D Criterion A + E	Criterion A + B Criterion A + C Histology shows the presence of lymphoplasmacytic sclerosing pancreatitis in the resected pancreas

**Table 5:** Cons and pros of different kinds of imaging.

Imaging	Imaging findings	Advantage	Disadvantage	When to select
US	Diffuse enlargement, hypoechoic pancreas	Low price, noninvasive, and easy to operate	Lack of specificity	Physical examination

CT	Diffuse morphological pancreatic parenchymal enlargement, focal enlargement of the pancreas [72]	Being noninvasive, being easy to operate, high quality image for pancreatic parenchymal enlargement, differentiating AIP from pancreatic cancer	Less sensitivity in the pancreatic and bile duct lesion than MRCP and MRI	Evaluate the pancreatic parenchyma and differentiate AIP from pancreatic cancer

MRI	Hypointense signal on T1 weighted images and relatively T2 hyperintense signal [75]	Being noninvasive, being easy to operate, showing the pancreatic fibrosis	Less sensitivity in pancreatic parenchymal than CT	Evaluate the pancreatic parenchyma

MRCP	Diffused narrow or segmental stenosis of main pancreatic ducts, the pancreatic segment of common bile duct stricture, proximal bile duct dilation, and gallbladder enlargement [80]	Being noninvasive, being easy to operate, presenting the pancreatic duct and bile duct and their relationship	Less sensitivity in the focal lesion of pancreatic parenchymal than CT	Evaluate the bile duct, pancreatic duct, and bile duct stricture

ERCP	Diffuse, irregular narrowing of the MPD [82, 83]	Diagnosis and treatment simultaneously, especially in the case of jaundice	Invasive	Evaluating the bile duct, pancreatic duct, and bile duct stricture, treatment for jaundice

EUS-FNA	—	Get the tissue with much less wound than surgery	Invasive May not get adequate tissue	Get the pancreatic tissue sample

PET	Uptake of fluorodeoxyglucose in organs other than the pancreas [88, 89]	Other organ involvement is easily detected	Expensive	Assess the other organ involvement, exclude malignant tumor

**Table 6:** Management strategy of AIP based on immunology therapy.

Time	0–12 weeks	12 weeks–6 months	6 months–3 years
Japan and Asian countries [94]			
Objective	Induction of remission	Maintenance therapy	
Drug	Prednisolone	Prednisolone	
Dose	0.6 mg/Kg/day for 2–4 weeks, tapered by 5 mg every 1-2 weeks to a maintenance dose	2.5–5.0 mg/day	

American and European countries [19]			
Objective	Induction of remission	Maintenance therapy	Observation
Drug	Prednisolone	Prednisolone	Immunomodulator/rituximab (when relapsing)
Dose	30–40 mg/day for 2–4 weeks, tapered by 5 mg every 1-2 weeks to a maintenance dose	5.0–7.5 mg/day	Undetermined
